# Variability and evolutionary implications of repetitive DNA dynamics in genome of *Astyanax scabripinnis* (Teleostei, Characidae)

**DOI:** 10.3897/CompCytogen.v11i1.11149

**Published:** 2017-03-06

**Authors:** Patrícia Barbosa, Eliza Viola Leal, Maelin da Silva, Mara Cristina de Almeida, Orlando Moreira-Filho, Roberto Ferreira Artoni

**Affiliations:** 1 Programa de Pós-Graduação em Genética Evolutiva e Biologia Molecular, Universidade Federal de São Carlos, Rodovia Washington Luís Km 235, São Carlos, SP, 13565-905, Brazil; 2 Programa de Pós-Graduação em Biologia Evolutiva, Universidade Estadual de Ponta Grossa, Avenida Carlos Cavalcanti 4748, Ponta Grossa, PR, 84030-900, Brazil

**Keywords:** Microsatellites chromosomic mapping, sex-specific GATAn sequence location, B chromosomes microsatellites accumulation

## Abstract

DNA sequences of multiple copies help in understanding evolutionary mechanisms, genomic structures and karyotype differentiation. The current study investigates the organization and distribution of different repetitive DNA in the standard complement and B chromosomes in *Astyanax scabripinnis* (Jenyns, 1842) chromosomes from three allopatric populations in Campos do Jordão region, São Paulo State, Brazil. The location of microsatellite sequences showed different chromosome distribution between Lavrinha Farm Stream (LFS) and Lake of Pedalinho (LP) populations. However, the karyotype of these populations basically followed the pattern of dispersed distribution in the A complement, conspicuous in telomeric/interstitial regions and preferential accumulation in the B chromosome. The B chromosome showed heterogeneous location of microsatellite probes CA, CAC and GA. The H3 and H4 histone genes were isolated from the total genome of the species and then the chromosomal mapping was performed by fluorescence *in situ* hybridization (FISH). The FISH signals showed high similarity for the probes H3 and H4 mapping in genomes of the populations analyzed. The sequences (GATA)*_n_* revealed a sex-specific trend between the chromosomal location in males and females at (LFS) and (LP) populations. Although species that comprise the *Astyanax scabripinnis* complex do not have morphologically differentiated sex chromosomes, the preferential GATA location – sex-associated – may represent a sex chromosome in differentiation.

## Introduction

DNA sequences of multiple copies help in understanding evolutionary mechanisms, genomic structures and karyotype differentiation, including in fishes. *Astyanax scabripinnis* (Jenyns, 1842) is a freshwater fish species of the family Characidae (Lima et al. 2003). It is widely distributed and recognized as a complex of cryptic species (Moreira-Filho and Bertollo 1991).

*Astyanax scabripinnis* is a model in evolutionary studies due to the frequent presence of B chromosomes in some populations (see Moreira-Filho et al. 2004 for review). Differences in the C-band pattern of this extra genome chromosome may be found and they vary from fully (Vicente et al. 1996) to partially heterochromatic (Mizoguchi and Martins-Santos 1997). Although a fully heterochromatic B chromosome comparable in morphology and size was found in the species *Astyanax fasciatus* (Cuvier, 1819), *Astyanax schubarti* (Britski, 1964) and *Astyanax scabripinnis* (Jenyns, 1842) (Moreira-Filho et al. 2001), the origin of these chromosomes is still a question that deserves attention. The occurrence of B microchromosomes, e.g. in *Astyanax goyacensis* (Santos et al. 2013), suggests the recurrent origins of these supernumerary chromosomes in *Astyanax* Baird & Girard, 1854. The most accepted hypothesis is that the B chromosome emerged in *Astyanax scabripinnis* from standard karyotype (subtelocentric or acrocentric chromosome) and it formed an isochromosome followed by heterochromatinization (Vicente et al. 1996, Mestriner et al. 2000, Vicari et al. 2011). However, there is no evidence that B chromosome is functional in this species, although recent studies show the *in situ* location of possible genes (sequences/probes) such as 18S rDNA and H1 histone in the B chromosome of *Astyanax paranae* – a species that belongs to complex *Astyanax scabripinnis* (Silva et al. 2014). The absence of activity in the 18S sequences, located in B chromosome, suggests that these genes are possible pseudogenes (Ferreira-Neto et al. 2012). However, the occurrence of functional genes in B chromosomes has been evidenced, mainly in plants (see Banaei-Moghaddam et al. 2015 for review).

Some functional genes found in the DNA of eukaryotes may have simple copies and unique sequences, whereas other genes have repetitive nature when they are found in more than one copy (Hardman 1986) such as in the case of multigenic families encoding the histones (Nei and Rooney 2005) and the rRNA (Martins 2007). These sequences are broadly conserved among the organisms and became important tools for evolutionary studies. The H3 and H4 histones are some of the most conserved proteins in the genome of eukaryotes, wherein the chromosomal location of H3 sequences varied among the organisms and it was already identified as: dispersed or tandem (Rooney et al. 2002). The rRNA may be classified as minor rDNA (5S), which is transcribed to rRNA 5S; and major rDNA (45S), which encodes the 28S, 5.8S and 18S rRNA (Eickbush and Eickbush 2007).

The sequences of repetitive DNA with tandem distribution are classified as satellites, microsatellites and minisatellites according to the degree of repetition (Jurka et al. 2005). The short in tandem repeated sequences of telomeric DNA (TTAGGG)*_n_* vary in number of copies in different organisms and contribute for the stability and replication of the chromosome (Blackburn and Szostak 1984).

Other repetitive DNAs with great evolutionary and functional interest are the *Bkm* (banded krait minor satellite) satellite sequences, which are found in the heterogametic sex of most vertebrates (Nanda et al. 1992). It consists of a simple GATA sequence repetition, which is a conserved tetranucleotide related to sex differentiation (Singh et al. 1984). Subramanian et al. (2003) assumed that the (GATA)*_n_* repetition plays a functional role in human sex chromosomes.

The microsatellite chromosomic mapping is widely used in evolutionary studies, including in *Astyanax* (Piscor and Parise-Maltempi 2016). The (GATA)*_n_* repetitive sequence, for example, allowed to verify differences in the chromosome structure and possible relation with the sexual differentiation in two species of lizards. (Pokorná et al. 2011).

The aim of this study is to investigate patterns of organization and distribution of different repetitive DNAs, in the standard complement and B chromosomes in *Astyanax scabripinnis*.

## Material and methods

### Animals and chromosome preparation

Fifty six specimens of *Astyanax scabripinnis* (18 females and 38 males) were analyzed from three different locations in the Campos do Jordão region, State of São Paulo, Brazil, collected with permission from Ministério do Meio Ambiente, Instituto Brasileiro do Meio Ambiente e dos Recursos Naturais Renováveis, Instituto Chico Mendes de Conservação da Biodiversidade – ICMBio MMA / IBAMA / SISBIO, number 15115-1: Lavrinha Farm Stream (LFS) (22°40'49.5"S; 45°23'31.9"W), Lake of Pedalinho (LP) (22°43'02.8"S; 45°33'91.9"W) and Ribeirão das Perdizes (RP) (22°44'35.3"S; 45°34'11.6"W). For cytogenetic analyses, the specimens were anesthetized with benzocain 0.01% and dissected, and the mitotic chromosomes were obtained from kidney tissue using the technique described by Bertollo et al. (1978) with modifications (Blanco et al. 2012). The C-banding technique was performed following the protocol described by Sumner (1972) and nucleolar organizing regions (Ag-NORs) was employed according to Howell and Black (1980). The chromosomes were classified as metacentric (m), submetacentric (sm), subtelocentric (st) and acrocentric (a), based on the classification proposed by Levan et al. (1964). All procedures were made according to international protocols for animal testing and authorized by the Ethic Committee in Animal Experimentation (protocol number 4509/08) of Universidade Estadual de Ponta Grossa. The specimens were identified and received a deposit number from Coleção Ictiológica do NUPELIA (Núcleo de Pesquisas em Limnologia, Ictiologia e Aquicultura) of Universidade Estadual de Maringá (NUP number 17482, 17484, 17486).

### DNA amplification and sequencing

The genomic DNA extraction was carried out by the cetyl trimethyl ammonium bromide method (Murray and Thompson 1980) with modifications. The primers used in PCR are described in Table 1. To obtain the 18S rDNA probe, 18S primers isolated from *Prochilodus argenteus* Spix & Agassiz, 1829 were used according to Hatanaka and Galetti Jr (2004). For the 5S rDNA, primers isolated from the rainbow trout were used according to Martins and Galetti Jr (1999). The *As*51 satellite DNA described by Mestriner et al. (1999) was obtained from the nuclear DNA of *Astyanax scabripinnis*. The (TTAGGG)*_n_* and (GATA)*_n_* probes amplification followed Ijdo et al. (1991). The H3 histone gene amplification followed Colgan et al. (1998) and H4 histone gene amplification followed Pineau et al. (2005). The PCR assay were performed using 100 ng DNA template in a final volume of 25 μl. Each reaction contained 1 X PCR buffer, 1.5 mM MgCl_2_, 200 μM dNTPs, 0.1 μM of each primer (10 pmol), and 0,5 U *Taq* DNA polymerase (Invitrogen). PCR of histone sequences was performed in the Eppendorf Mastercycler (30 cycles of 5 min at 95°C, 30 s at 95°C, 45 s at 52°C, 1 min 20 s at 72°C, and 7 min at 72°C). The product purification of the target DNA was performed using the High PCR Cleanup Micro Kit (GE Healthcare Amersham Biosciences^TM^), following manufacturer instructions.

**Table 1.** Primer sequences employed.

Chromosomal markers	Primer sequence (5’-3’)	Reference
**18S rDNA F**	GTAGTCATATGCTTGTCTC	Hatanaka and Galetti 2004
**18S rDNA R**	TCCGCAGGTTCACCTACGGA	Hatanaka and Galetti 2004
**5S rDNA F**	TACGCCCGATCTCGTCCGATC	Martins and Galetti 1999
**5S rDNA R**	CAGGCTGGTATGGCCGTAAGC	Martins and Galetti 1999
***As*51 F**	GGTCAAAAAGTCGAAAAA	Mestriner et al. 1999
***As*51 R**	GTACCAATGGTAGACCAA	Mestriner et al. 1999
**H3 F**	ATGGCTCGTACCAAGCAGACVGC	Colgan et al. 1998
**H3 R**	ATATCCTTRGGCAT RATRGTGAC	Colgan et al. 1998
**H4 F**	TSCGIGAYAACATYCAGGGIATCAC	Pineau et al. 2005
**H4 R**	CKYTTIAGIGCRTAIACCACRTCCAT	Pineau et al., 2005

The nucleotide sequencing of the clones and DNA fragments was performed in an automatic sequencer ABI 3130x1, using o Kit Big Dye (Applied Biosystems) following manufacturer instructions. The sequences were aligned in Clustal W program (Thompson et al. 1994), using the BioEdit 7.0 editor (Hall 1999). To verify the identity, the sequences were subjected to search in BLASTn (http://www.ncbi.nlm.nih.gov/blast) and deposited in the GenBank (http://www.ncbi.nlm.nih.gov/genbank/) with the following accession number: H3 histone gene (LFS – KT633503, LP – KT633504, RP – KT633505), H4 histone gene (LFS – KT633506, LP – KT633507, RP – KT633508).

### Chromosome probe

Seven different types of repetitive probes were used as chromosomal markers: 18S rDNA; 5S rDNA; *As*51 satellite DNA; H3 and H4 histone genes; the general vertebrate telomere sequence minisatellite (TTAGGG)*_n_*; and the (GATA)*_n_* sequence. The *As*51 satellite DNA, 5S rDNA and H4 histone gene probes was labelled with Biotin-11-dUTP (Roche Applied Science), whereas 18S rDNA, H3 histone gene, (GATA)*_n_*, and (TTAGGG) *_n_* probes were labelled with digoxigenin by *nick translation*.

Oligonucleotide probes containing microsatellite sequences (CA)_15_, (CAC)_10_, (CAG)_10_, (CAT)_10_, (GA)_15_, (GAA)_10_, (GAG)_10_ and (GC)_15_ were directly labeled with Cy5 during synthesis by Sigma (St. Louis, MO, USA), as described by Kubat et al. (2008).

### Fluorescence in situ hybridization (FISH)

For each FISH assay 30 cells were analyzed. The FISH was performed using the protocol of Pinkel et al. (1986) with adaptations. The protocol was adjusted to high stringency (2.5 ng/mL probe, 50% deionized formamide, 10% dextran sulphate, 2 × SSC at 37°C overnight). After hybridization, the slides were washed in 15% formamide/0.2 × SSC at 42°C for 20 min and 4 × SSC/0.05% Tween at room temperature for 10 min. The signal detection was performed using alexa fluor 488 streptavidin (Molecular Probes^TM^) for 5S rDNA and H4 histone gene, whereas the anti-digoxigenin-rhodamin (Roche^TM^) was used for 18S rDNA, H3 histone gene, (GATA)*_n_* and (TTAGGG)*_n_* probes detection. The chromosomes were counterstained with DAPI (0.2 µg/mL) diluted in antifade solution (Fluka^TM^). Chromosomes were analyzed under epifluorescence microscopy Zeiss AxioCam MRm and software ZEN pro 2011 (Carl Zeiss^®^).

## Results

### Chromosomal analysis and rDNA

**Figure 1.**
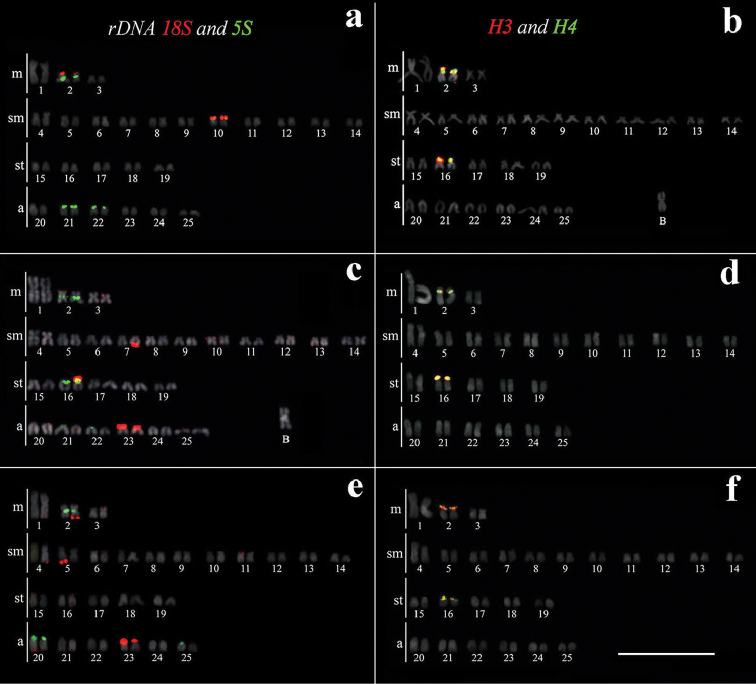
Karyotype of the three *Astyanax scabripinnis* populations (**a**, **b** Lavrinha Farm Stream – LSF); (**c**, **d** Lake of Pedalinho – LP); (**e**, **f** Ribeirão das Perdizes – RP). In **a**, **c**, **e** are visualized signals of 18S (red) and 5S (green) with rDNA probes and in **b**, **d**, **f** H3 (red) and H4 (green) with histones probes, after double Fluorescence *in situ* Hybridization (FISH), respectively. Scales bar: 10 µm.

The diploid number verified for *Astyanax scabripinnis* was 2n = 50 chromosomes in the three analyzed populations. The fundamental number (FN) was equal to 88 and karyotype formula composed by 6 m + 22 sm + 10 st + 12 a. Some specimens showed B chromosomes in 100% of the analyzed cells (2n = 51) (Suppl. material 1). These data corroborated previous studies in this species complex (see Moreira-Filho et al. 2004 for review). The (LFS) population showed 18S rDNA sites in the metacentric chromosome pair No. 2 and submetacentric pair No. 10 (carrier of Ag-NOR), both in short arm in terminal position The 5S rDNA sites was syntenic with 18S rDNA was located in the chromosome pair No. 2 in proximal region. In addition, plus sites were found in the acrocentric chromosomic pairs No. 21 and 22 (Fig. 1a). In the (LP) population the 18S rDNA was in terminal regions of pairs 7 sm, 16 st (carrier of Ag-NOR) and 23 a, since the location occurs in only one of the homologues in the pairs 7 and 16 (Fig. 1c). The 5S rDNA showed signal in the pair 2 m in proximal region and in the pair 16 st in terminal region, co-located with 18S rDNA (Fig. 1c). The 18S rDNA in the (RP) population was also found in only one of the homologues in the pairs 2 m and 5 sm (carrier of Ag-NOR) and pair 23 a, whereas 5S rDNA was located in the pair 2, in proximal region and syntenic to 18S rDNA and in the pair 20 a (Fig. 1e). The B chromosome did not evidence signal with the ribosomal probes used (Fig. 1c).

### H3 and H4 histone genes and sequence analysis

The amplification of the H3 and H4 histone genes from the total genome of the specimens was performed. The product of the PCR generated bands with approximately 400 base pair (bp), when it was analyzed in gel electrophoresis (Suppl. material 2). This product was submitted to nucleotide sequencing and it was used as FISH probe. The sequence alignment of H3 and H4 histones with other sequences of *Astyanax* deposited in GenBank resulted in 97% minimum similarity.

The H3 and H4 histones were co-located in two chromosomal pairs: metacentric pair No. 2 and subtelocentric pair No. 16 in the three populations (LFS, LP, RP). The sites of chromosome metacentric pair 2 were proximal, whereas the signal were terminal in other chromosomal pairs (Figs 1b, d, f). The B chromosome did not show any signal with the used probes H3 and H4 (Fig. 1b).

### (TTAGGG)_n_ sequence, (GATA)_n_ sequence and heterochromatin localization

The probe of the minisatellite sequence (TTAGGG)*n* was uniformly located in the telomeres of all chromosomes at the studied populations, including B chromosome. No interstitial signal (ITS) was found (data not shown) (Suppl. material 3).

**Figure 2.**
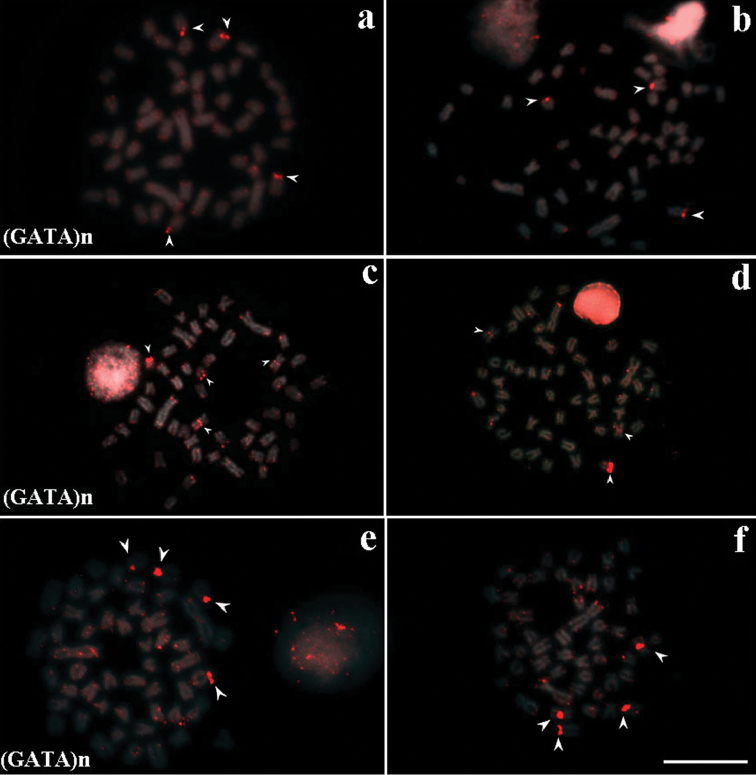
Fluorescence *in situ* Hybridization in metaphases of the three *Astyanax scabripinnis* populations (**a**, **b** Lavrinha Farm Stream – LSF); (**c**, **d** Lake of Pedalinho – LP); (**e**, **f** Ribeirão das Perdizes – RP) with (GATA)n probe. **a**, **c**, **e** males and **b**, **d**, **f** females. Scales bar: 10 µm

**Figure 3.**
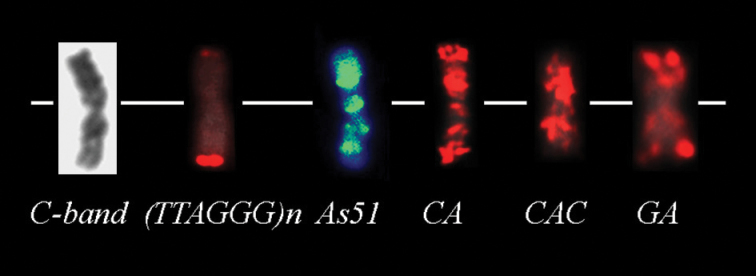
B chromosome of *Astyanax scabripinnis* showing the chromatin and localization of DNA sequences by FISH.

The (GATA)*_n_* sequence was hybridized in males and females of the three populations (Fig. 2), and it was followed by C-banding for the analysis of heterochromatic regions (data not shown) (Suppl. material 4). The acrocentric chromosome pairs 20 and 21 showed signal in centromeric regions of male cells in (LFS) (Fig. 3a). The non-homologous chromosomes 21 and 23 bear (GATA)n signal in centromeric region of females, as well as in terminal long arm region of a chromosome of subtelocentric pair No. 15 (Fig. 3b). All the regions hybridized with the (GATA)*_n_* probe overlapped the positive C-band regions.

Males and females from the (LP) population did not show significant differences regarding the signal of the (GATA)*_n_* probe. Females showed three main signals: the homologues of metacentric chromosome pair No. 2, in the proximal region, without heterochromatin; and one of the homologues of pair 24 a in terminal region of the long arm, with evidences of heterochromatin (Fig. 2d). Males showed the same signal, with an additional signal in one of the homologues of pair 24 a (Fig. 2c), without heterochromatin accumulation.

The male specimens from the (RP) population showed the pair of subtelocentric chromosomes 15 and 16 as their main signal (Fig. 2e), whereas the (GATA)*_n_* signals on females is in the pair 2 m in proximal region and in the pair 20 a in terminal region (Fig. 2f) – heterochromatin places are associated with the probe in both sexes. The (GATA)*_n_* probe did not evidence signal in the B chromosomes (Fig. 2a).

### As51 satellite DNA and / microsatellite sequences probes

The *As*51 satellite DNA occurs in acrocentric chromosomes of the three populations: pairs 22 and 23 in the (LFS) and (LP) populations, pair 20 in the (RP) population and also in the chromosome B (Fig. 3). According to Barbosa et al. (2015) (Fig. 5), additional signals are observed in pairs 4 and 6 sm of the (LFS) population.

**Figure 4.**
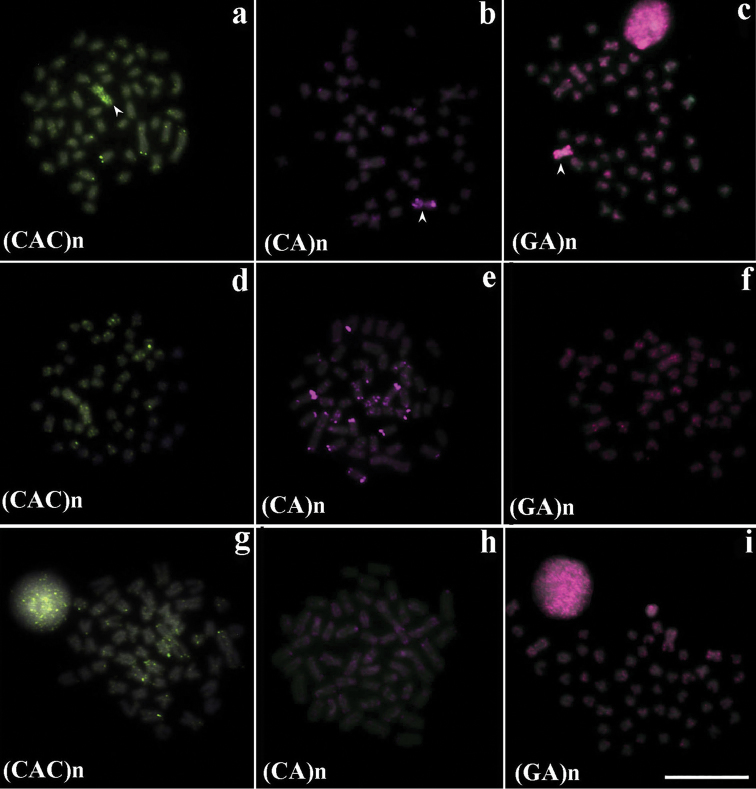
Fluorescence *in situ* Hybridization in metaphases of the three *Astyanax scabripinnis* populations (**a, b, c** Lavrinha Farm Stream – LSF); (**d**, **e**, **f** Lake of Pedalinho – LP); (**g, h, i** Ribeirão das Perdizes - RP) with microsatellites probes. Scales bar: 10 µm.

The oligonucleotide probes (CAC)_10_, (CA)_15_ and (GA)_15_ microsatellite sequences showed dispersed signal among the chromosomes of complement A in the three populations. In addition, the probes showed an accumulation in B chromosomes of individuals of the (LFS) population (Fig. 3 and Fig. 4a, b, c). However, the (CA)_15_ sequence revealed preferential localization on telomeric regions and on some interstitial sites in the chromosomes of the (LP) population, although this distribution was not homogeneous among the chromosomes (Fig. 4d, e, f). In contrast, the others tested microsatellites probes: (CAG)_10_, (CAT)_10_, (GAA)_10_, (GAG)_10_ and (GC)_15_ appeared to be dispersed and poorly represented in the karyotypes of *Astyanax scabripinnis* (data not shown).

**Figure 5.**
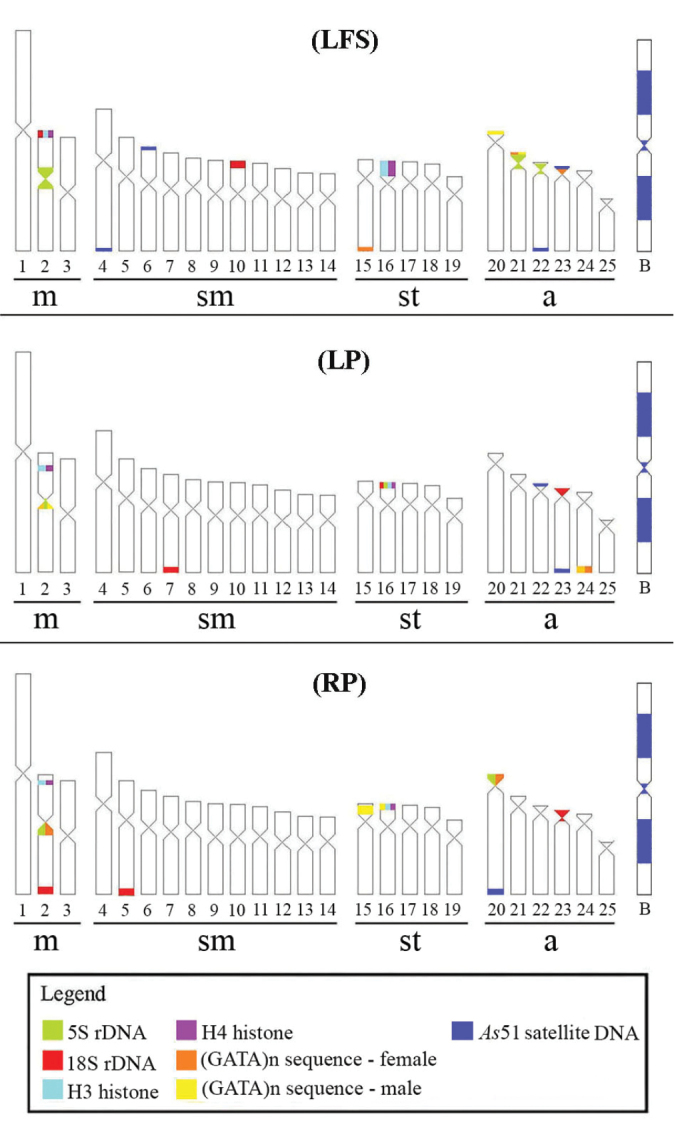
Idiograms of the three *Astyanax scabripinnis* populations (LFS: Lavrinha Farm Stream); (LP: Lake of Pedalinho); (RP: Ribeirão das Perdizes) highlighting all markers of molecular cytogenetics used in this study.

## Discussion

Although the diploid number (2n) verified for *Astyanax scabripinnis* species complex indicates a variation of 46, 48 or 50 chromosomes (Moreira-Filho and Bertollo 1991), data found for Campos do Jordão region showed a common feature regarding the diploid and fundamental numbers (2n = 50; FN = 88). These fishes keep a polymorphic B chromosome in some populations (Vicente et al. 1996, Ferro et al. 2003, Vicari et al. 2011, present study). The results obtained herein for the karyotype of the (LFS) population corroborated the findings by Ferro et al. (2001), but differentiated by the verification of a single NOR without the occurrence of multiple sites. It may represent the fixation of only one chromosomal pair carrying 18S ribosomal cistrons in this isolated population.

The karyotype description for the (LP) population was performed here for the first time. The population showed standard diploid number and karyotype formula, with multiple NORs. The karyotype of the (RP) population corroborates data obtained by Vicente et al. (1996) regarding the presence of B chromosome. However, differences in the location of NORs within this population were also observed, since they were exclusively located in the short arm of pair 10 sm in previous studies. Probably, these trends are mediated by the association of additional sites of NORs with *LINE* retrotransposons – among other possible transposable elements (TEs) – and with repetitive sequences such as *As*51 (Barbosa et al. 2015).

*Astyanax scabripinnis* forms small isolated populations in streams and it facilitates the establishment of chromosomal changes that lead to polymorphism which may explain karyotype variability among individuals and populations (see Moreira-Filho et al. 2004 for review). The higher amount in the number of ribosomal gene copies and in the transposition may originate natural polymorphisms, e.g. in *Gymnotus carapo* Linnaeus, 1758, *Apteronotus albifrons* (Linnaeus, 1766), *Sternopygus macrurus* (Bloch & Schneider, 1801), *Eigenmannia virescens* (Valenciennes, 1836) and *Eigenmannia* sp., in which only one of the homologues carries the NOR (Foresti et al. 1981), as it occurs in the specie *Astyanax scabripinnis*. Variations in NORs location may be also due to structural chromosomal rearrangements, such as translocation of ribosomal sites and/or inversions (Gornung 2013) or by association with TEs (reviewed in Reed and Philips 2000). Data available for the detection and location of major ribosomal DNA genes in different populations of *Astyanax scabripinnis* make the evolutionary forces possible to generate, fix and diversify the NORs. One hypothesis is that the balance would happen due to evolutionary processes – opposite from ‘birth-and-death’ – *versus* the concerted evolution.

The number of 5S rDNA sites varied between 4 and 5 in the populations, wherein the proximal signal located in pair 2 m is a feature conserved among them. Similar results were described by Ferro et al. (2001) and Mantovani et al. (2005) in a study involving *Astyanax scabripinnis* and by Silva et al. (2014) regarding *Astyanax paranae*. The conserved location of 5S rDNA proximal to the centromere seems to provide protection against rearrangements and dispersions in chromosomes (Martins and Wasko 2004). However, the increase in the number of sites may be associated to TEs, as it occurs in *Gymnotus paraguensis* Albert & Crampton, 2003 (Silva et al. 2011), or to pseudogenes, as suggested for *Semaprochilodus taeniurus* (Valenciennes, 1821) (Terencio et al. 2012), fact that is frequently associated to the weaknesses of the genome rich in repetitive sequences (Rebordinos et al. 2013).

Data on the location of histone genes are still rare. According to Pendás et al. (1994), these genes are arranged in tandem in a unique chromosomal pair. The chromosome mapping of H3 and H4 probes sequences in *Astyanax scabripinnis* was similar to verified by Silva et al. (2013) in *Astyanax bockmanni* and Silva et al. (2014) in Astyanax paranae, which are in syntenic organization in the short arm of metacentric chromosome pair No. 2. Thus, we suggest a conserved location among the species of the genus. The co-location of these clusters is also found in other evolutionarily distant organisms, such as in Acrididae grasshoppers (Cabrero et al. 2009) and it increases the evidences regarding the syntenic positional conservation of these sequences. However, exceptions must be highlighted, as the multiple sites of H2B-H2A and H3 histones found in the marine perciform *Rachycentron canadum* (Linnaeus, 1766) (Costa et al. 2014). Thus, it is still necessary to expand the studies on the physical location and organization of histone genes, in order to better understand this evolutionary scenario.

The location of interstitial telomeric sites (ITS) is an important tool that helps to tell the evolutionary history of a group (Meyne et al. 1990) and it indicates the occurrence of possible chromosomal rearrangements (Ashley and Ward 1993). Centric fusions in chromosomes are described in fish (see Ocalewics 2013 for review), for example, in the Loricariidae family, *Rineloricaria lima* (Rosa et al. 2012) and *Hypostomus iheringii* (Traldi et al. 2013). So far, there is no evidence of ITS in *Astyanax scabripinnis* using telomeric probe. According to the study, the signal was homogeneous in terminal regions of both chromosomal arms of complement A and also in chromosome B, and it suggests that possible rearrangements (centric fusions and/or paracentric inversions) able to lead to interstitial locations of ITS are not frequent, or even that interstitial telomeric sequences are quickly eliminated from the genome of these fishes.

Furthermore, an accumulation of repetitive sequences in the chromosome 2 of the three studied populations and also in the chromosome 16 of the (LP) population was evident. Piscor and Parisi-Maltempi (2016) proposed evolutionary pathways for microsatellites in the genome of *Astyanax*. The authors propose that the association of 5S rDNA-GATA can stabilize the structure of DNA and act as ‘hot spots’ for chromosomal recombination. Our data contrast with the Piscor and Parisi-Maltempi (2016) and show that GATA sequences are not restricted to co-location with the 5S rDNA and may occur with sex-specific location in *Astyanax scabripinnis*.

The location of (GATA)*_n_* repetitive sequences revealed a slightly dispersed pattern in chromosomes of *Astyanax scabripinnis*, but with preferential accumulation in terminal and interstitial regions of the three studied populations. Dispersed signal of GATA sequences in fishes are verified by FISH in *Solea senegalensis* Kaup, 1858 (Cross et al. 2006), as well as in species of genus *Hypostomus* Lacépède, 1803 (Traldi et al. 2013) and are conspicuous in a unique chromosomal pair in *Halobatrachus didactylus* Bloch & Schneider, 1801 (Merlo et al. 2007). Jones and Singh (1985) assumed that sequences of the family *Bkm* – the major component of the satellite DNA isolated of snakes by Epplen et al. (1982) – tend to accumulate over the sex chromosomes W and Y of the organisms. Afterwards, Subramanian et al. (2003) suggested that the (GATA)*_n_* sequences have gradually accumulated in the genome of the organisms in the evolutionary scale and they could regulate the expression of close genes by means of chromatin reorganization. This last hypothesis favors the explanation for the widely dispersed location of (GATA)*_n_* sequences over fish chromosomes, but it raises a suspicion of link with sex determination in the case of *Astyanax scabripinnis*, wherein the location of these sequences by FISH appears to be sex-specific. It is worth highlighting that (GATA)*_n_* sequences are rarely coincident to heterochromatic regions in the *Astyanax scabripinnis* populations studied herein.

The presence of B chromosome in *Astyanax scabripinnis* confirmed the heterochromatic pattern found in different populations (see Moreira-Filho et al. 2004 for review). However, the (GATA)*_n_* sequence, ITS and moderately repetitive sequences such as histones and ribosomal DNAs were not identified in these supernumerary chromosomes. Nevertheless, the location of microsatellite sequences ((CA)*_n_*; (CAC)*_n_*; (GA)*_n_*) showed different patterns and it suggests the heterogeneous nature of the chromatin for B chromosomes studied herein.

The mapping of microsatellites in the chromosomes may help in investigating the chromatin nature. Similarly, to data found in chromosomes of *Astyanax scabripinnis* (LP), there might be similar patterns among different species, such as the dinucleotide (CA)_15_, which is found at the centromeric region of chromosome W in lizards *Eremias velox* Eremchenko & Panfilov, 1999 (Pokorná et al. 2011) and in telomeric regions with some interstitial sites in *Erythrinus erythrinus* Bloch & Schneider, 1801 (Yano et al. 2014). The *As*51 satellite DNA is presented in large pericentromeric distal blocks when it is hybridized over B chromosome in this specie (Mestriner et al. 2000, Vicari et al. 2011). As it was described by Mestriner et al. (2000), the repetitive DNA of the complex *Astyanax scabripinnis* by 59% AT bases. In contrast, the current study evidenced the preferential accumulation of dinucleotide (CA)_15_ and (GA)_15_ and the trinucleotide (CAC)_10_ for B chromosome. The compartmentalization of microsatellites may also be verified in the heteromorphic W sex chromosomes of *Triportheus* Cope, 1872 and *Leporinus* Spix & Agassiz, 1829 (Cioffi et al. 2012, Poltronieri et al. 2014). These evidences of microsatellite sequences organization in heterochromatinized chromosomes and non-recombinant suggest the involvement of this DNA class in the amplification and differentiation of these chromosomes.

Thus, the study brings up the required association condition and co-location of different group of repetitive DNAs expressed and non-encoding. Although these sequences may be related to the evolution of genome and karyotype, they still suggest a possible relation with sex determination in the species.

## Supplementary materials

Supplementary material 1 Karyotype data of the three Astyanax aff. scabripinnis populations analyzed: diploid number, karyotype formulae, Ag-NORs and repetitive DNAs locations Data type: karyotype data This dataset is made available under the Open Database License (http://opendatacommons.org/licenses/odbl/1.0/). The Open Database License (ODbL) is a license agreement intended to allow users to freely share, modify, and use this Dataset while maintaining this same freedom for others, provided that the original source and author(s) are credited.Patrícia Barbosa, Eliza Viola Leal, Maelin da Silva, Mara Cristina de Almeida, Orlando Moreira-Filho, Roberto Ferreira Artoni

Supplementary material 2 DNA sequences of the histone PCR product of the three Astyanax aff. scabripinnis populations employed as probes for FISH in this study Data type: molecular data Explanation note: LFS: Lavrinha Farm Stream; LP: Lake of Pedalinho; RP: Ribeirão das Perdizes This dataset is made available under the Open Database License (http://opendatacommons.org/licenses/odbl/1.0/). The Open Database License (ODbL) is a license agreement intended to allow users to freely share, modify, and use this Dataset while maintaining this same freedom for others, provided that the original source and author(s) are credited.Patrícia Barbosa, Eliza Viola Leal, Maelin da Silva, Mara Cristina de Almeida, Orlando Moreira-Filho, Roberto Ferreira Artoni

Supplementary material 3 Fluorecence in situ Hybridization in metaphases of the three Astyanax aff. scabripinnis populations with telomeric probe (TTAGGG)n Data type: karyotype data Explanation note: a: Lavrinha Farm Stream – LSF, b: Lake of Pedalinho – LP, c: Ribeirão das Perdizes – RP. This dataset is made available under the Open Database License (http://opendatacommons.org/licenses/odbl/1.0/). The Open Database License (ODbL) is a license agreement intended to allow users to freely share, modify, and use this Dataset while maintaining this same freedom for others, provided that the original source and author(s) are credited.Patrícia Barbosa, Eliza Viola Leal, Maelin da Silva, Mara Cristina de Almeida, Orlando Moreira-Filho, Roberto Ferreira Artoni

Supplementary material 4 Metaphases of the three Astyanax aff. scabripinnis populations (a, d); (b, e); (c, f) showing C-banding. Staining with propidium iodide converted to grayscale Data type: karyotype data This dataset is made available under the Open Database License (http://opendatacommons.org/licenses/odbl/1.0/). The Open Database License (ODbL) is a license agreement intended to allow users to freely share, modify, and use this Dataset while maintaining this same freedom for others, provided that the original source and author(s) are credited.Patrícia Barbosa, Eliza Viola Leal, Maelin da Silva, Mara Cristina de Almeida, Orlando Moreira-Filho, Roberto Ferreira Artoni
