# Supplementary material for: Variability and evolutionary implications of repetitive DNA dynamics in genome of Astyanax scabripinnis (Teleostei, Characidae)
Source: Comp Cytogenet. 2017 Mar 6;11(1):143–62. doi: 10.3897/CompCytogen.v11i1.11149 (PMC5599702; doi:10.3897/CompCytogen.v11i1.11149)
Supplement: Supplementary material 1 — Karyotype data of the three Astyanax aff. scabripinnis populations analyzed: diploid number, karyotype formulae, Ag-NORs and repetitive DNAs locations [file comparative_cytogenetics-11-143-s001.docx]

| Chromosomal markers | Primer sequence (5’-3’) | Reference |
| --- | --- | --- |
| 18S rDNA F | GTAGTCATATGCTTGTCTC | Hatanaka and Galetti Jr., 2004 |
| 18S rDNA R | TCCGCAGGTTCACCTACGGA | Hatanaka and Galetti Jr., 2004 |
| 5S rDNA F | TACGCCCGATCTCGTCCGATC | Martins and Galetti Jr., 1999 |
| 5S rDNA R | CAGGCTGGTATGGCCGTAAGC | Martins and Galetti Jr., 1999 |
| *As*51 F | GGTCAAAAAGTCGAAAAA | Mestriner et al., 1999 |
| *As*51 R | GTACCAATGGTAGACCAA | Mestriner et al., 1999 |
| H3 F | ATGGCTCGTACCAAGCAGACVGC | Colgan et al., 1998 |
| H3 R | ATATCCTTRGGCAT RATRGTGAC | Colgan et al., 1998 |
| H4 F | TSCGIGAYAACATYCAGGGIATCAC | Pineau et al., 2005 |
| H4 R | CKYTTIAGIGCRTAIACCACRTCCAT | Pineau et al., 2005 |
