# Supplementary material for: Variability and evolutionary implications of repetitive DNA dynamics in genome of Astyanax scabripinnis (Teleostei, Characidae)
Source: Comp Cytogenet. 2017 Mar 6;11(1):143–62. doi: 10.3897/CompCytogen.v11i1.11149 (PMC5599702; doi:10.3897/CompCytogen.v11i1.11149)
Supplement: Supplementary material 2 — DNA sequences of the histone PCR product of the three Astyanax aff. scabripinnis populations employed as probes for FISH in this study [file comparative_cytogenetics-11-143-s002.docx]

**Table 2** Karyotype data of the three *Astyanax* aff. *scabripinnis* populations analyzed: diploid number, karyotype formulae, Ag-NORs and repetitive DNAs locations (*Reference: Barbosa et al., 2015)

| Location | Lavrinha Farm Stream (LFS) | Lake of Pedalinho (LP) | Ribeirão das Perdizes (RP) |
| --- | --- | --- | --- |
| 2n | 50/51 | 50/51 | 50/51 |
| Karyotype formula | 6m+22sm+10st+12a | 6m+22sm+10st+12a | 6m+22sm+10st+12a |
| Ag-NORs sites | 10sm; short arm | 16st; short arm | 5sm; long arm |
| 18S rDNA | 2m, short arm, terminal  10sm, short arm, terminal | 7sm, long arm, terminal  16st, short arm, terminal  23a, centromeric, terminal | 2m, long arm, terminal  5sm, long arm, terminal  23a, centromeric |
| 5S rDNA | 2m, proximal  21a, centromérica, terminal  22a, centromérica, terminal | 2m, proximal  16st, short arm, terminal | 2m, proximal  20a, centromeric |
| H3 and H4 histones | 2m, short arm, terminal  16st, short arm, terminal | 2m, short arm  16st short arm | 2m, short arm  16st, short arm |
| *As*51 satellite DNA | 4sm, long arm, terminal*  6sm, short arm, terminal*  22a, long arm, terminal*  23a, short arm, terminal*  equally-located pericentromeric regions in B chromosome* | 22a, centromeric  23a, long arm, terminal  equally-located pericentromeric regions in B chromosome | 20a, long arm, terminal  equally-located pericentromeric regions in B chromosome |
| (GATA)_n_ male | 20a, centromeric  21 a, centromeric | 2m, proximal  24a, long arm, terminal | 15st, short arm  16st, short arm |
| (GATA)_n_ female | 15st, long arm, terminal  21a, centromeric  23a, centromeric | 2m, proximal  24a, long arm, terminal | 2m, proximal  20a, centromeric |
