# Supplementary figures and images for: Variability and evolutionary implications of repetitive DNA dynamics in genome of Astyanax scabripinnis (Teleostei, Characidae)
Source: Comp Cytogenet. 2017 Mar 6;11(1):143–62. doi: 10.3897/CompCytogen.v11i1.11149 (PMC5599702; doi:10.3897/CompCytogen.v11i1.11149)

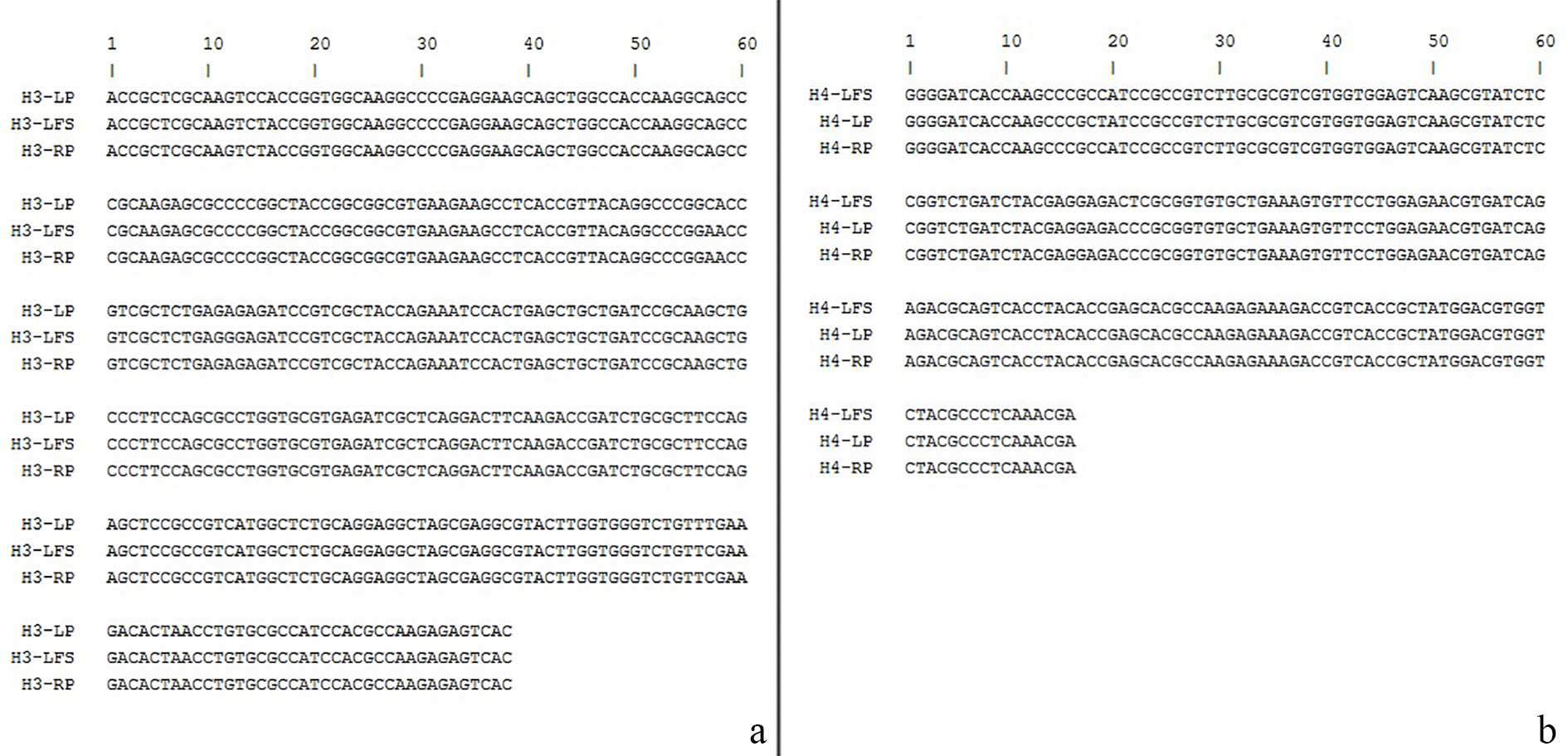

Supplement: Supplementary material 3 — Fluorecence in situ Hybridization in metaphases of the three Astyanax aff. scabripinnis populations with telomeric probe (TTAGGG)n [file comparative_cytogenetics-11-143-s003.jpg]

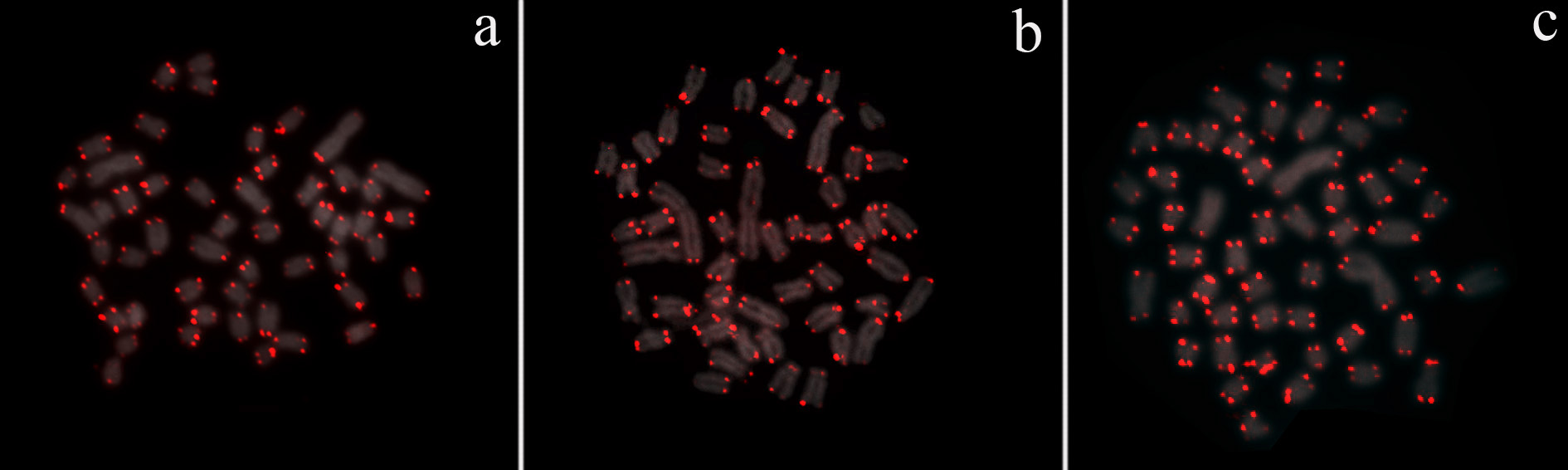

Supplement: Supplementary material 4 — Metaphases of the three Astyanax aff. scabripinnis populations (a, d); (b, e); (c, f) showing C-banding. Staining with propidium iodide converted to grayscale [file comparative_cytogenetics-11-143-s004.jpg]
